# Mitochondrial protein import stress augments α-synuclein aggregation and neural damage independent of bioenergetics

**DOI:** 10.21203/rs.3.rs-3136613/v1

**Published:** 2025-08-18

**Authors:** Xin Jie Chen, Liam Coyne, Arnav Rana, Xiaowen Wang, Sanaea Bhagwagar, Yumiko Umino, Eduardo Solessio, Frank Middleton

**Affiliations:** State University of New York Upstate Medical University; Johns Hopkins Hospital; State University of New York Upstate Medical University; State University of New York Upstate Medical University; State University of New York Upstate Medical University; SUNY Upstate Medical University; SUNY Upstate Medical University

## Abstract

Genetic and environmental factors are known to converge on mitochondria to cause Parkinson’s disease (PD). However, the mechanisms by which mitochondrial dysfunction contributes to neurodegeneration remain incompletely understood. Non-bioenergetic pathways of the mitochondria are increasingly appreciated, but confounding bioenergetic defects are a major barrier to experimental validation. Here, we show that mild mitochondrial protein import stress augments neural damage independent of bioenergetics. We induce protein import stress in a mouse model of PD expressing α-synuclein(A53T). The double mutant mice demonstrate increased size of α-synuclein aggregates, increased aggregation of mitochondrial preproteins, heightened neuroinflammation and worsened motor defect relative to α-synuclein(A53T) single mutants. Importantly, we found no evidence of bioenergetic defects in any of the mutant mice. These data suggest that mitochondrial protein import stress, which can be induced by many types of mitochondrial injuries, can contribute to neural damage through cytosolic proteostatic stress and possible co-aggregation of mitochondrial and neuropathogenic proteins independent of bioenergetics.

## Introduction

Mitochondrial defects and cytosolic protein aggregation in neurons are co-manifested across neurodegenerative diseases such as Parkinson’s disease (PD), Lewy Body Dementia, Alzheimer’s disease, Amyotrophic Lateral Sclerosis (ALS) and Huntington’s disease^1–4^. These two hallmarks are virtually ubiquitous despite significant variations in genetic and phenotypic makeup among diseases. While both hallmarks can individually drive neurodegeneration, whether and how they interact during initiation and/or progression of these complex diseases are poorly understood, and likely to be multifactorial. The effects of pathogenic cytosolic protein species on mitochondrial function have been extensively investigated^5^. By comparison, whether and how mitochondrial damage affects cytosolic proteostasis and pathological protein aggregation is vastly understudied.

Recent work in yeast and human cells has shown that a wide range of mitochondrial stressors can affect cell viability by reducing the import of mitochondrial preproteins causing their toxic accumulation and aggregation in the cytosol^6–12^. This cell stress mechanism was termed mitochondrial Precursor Overaccumulation Stress (mPOS)^13^. In the context of neurodegeneration, this raises the intriguing possibility that mitochondrial dysfunction could contribute to cytosolic protein aggregation, either by directly causing proteotoxicity or by amplifying preexisting proteostatic stress in the cytosol driven by misfolded pathogenic proteins (e.g., a-synuclein in PD)^14^. The critical barrier to testing this idea has been lack of an adequate animal model of non-lethal mitochondrial protein import stress, especially one that isolates the effect of protein import defects in the absence of confounding bioenergetic defects. Such a model is essential for testing the pathogenicity of protein import stress and mPOS.

Modeling mitochondrial protein import stress in animals is particularly challenging because virtually all mitochondrial functions, with some being essential for cell survival, depend on efficient import through a limited number of protein translocase channels. All but 13 of the 1,000–1,500 mitochondrial proteins are encoded in the nucleus, synthesized by cytosolic ribosomes, and then imported into the organelle. The import machinery is intricate, relying on numerous chaperones both inside and outside mitochondria, as well as multiple pore-forming protein complexes in the inner and outer mitochondrial membranes^15–17^. The sole entry point for >90% of mitochondrial proteins is the translocase of the outer membrane (TOM) complex. We previously engineered clinically relevant mutant variants of the yeast mitochondrial ADP/ATP carrier protein Aac2 and its human homolog ANT1 (or SLC25A4). We found that the mutant proteins can clog the mitochondrial protein import pathway and obstruct general protein import^18^. The best-characterized clogger mutant protein was the yeast Aac2p^A128P,A137D^ that has been shown to cause severe cell growth defect by preferentially clogging the TOM complex. Specifically, Aac2p^A128P,A137D^ accumulated at the TOM complex in cells and was unable to efficiently traverse the outer membrane in *in organello* protein import assay. In human cells, ANT1^A114P,A123D^, the equivalent of the yeast Aac2p^A128P,A137D^, also accumulated at the TOM complex and caused global retention of mitochondrial proteins in the cytosol. In a mouse model expressing *Slc25a4* ^*p.A114P,A123D*^ (“clogger” mice), we similarly found that unimported mitochondrial proteins accumulate in the cytosol of skeletal muscle, while mitochondrial respiration is only mildly affected. This correlates with a mild and age-dependent mitochondrial myopathy. We speculated that the mild phenotype is likely due to more rapid proteolytic clearance of the clogger protein in mammalian cells (present at ~0.1% of wild-type level). Interestingly, approximately 4% of the clogger mice become paralyzed around 12 months of age, suggesting a direct role of protein import clogging in neurodegeneration. However, the vast majority (~96%) of clogger mice have a normal lifespan, and do not develop overt neurological symptoms. Overall, our prior observations suggest that mitochondrial protein import is only mildly clogged in this model, presenting an opportunity to study the (patho)physiological effects of a mild reduction in mitochondrial protein import efficiency.

In the present study, we utilized clogger mice to study the specific effects of mild protein import stress on neurological function. Most importantly, we then used mouse genetics to test whether and how protein import stress might contribute to neural damage in a mouse model of PD that involves the cytosolic misfolding and aggregation of a mutant form of a-synuclein (A53T), that is a dominant cause of familial PD.

## Results

### Ant1^p.A114P, A123D^ does not affect mitochondrial bioenergetics, motor coordination or cognitive function.

**Figure 1:**
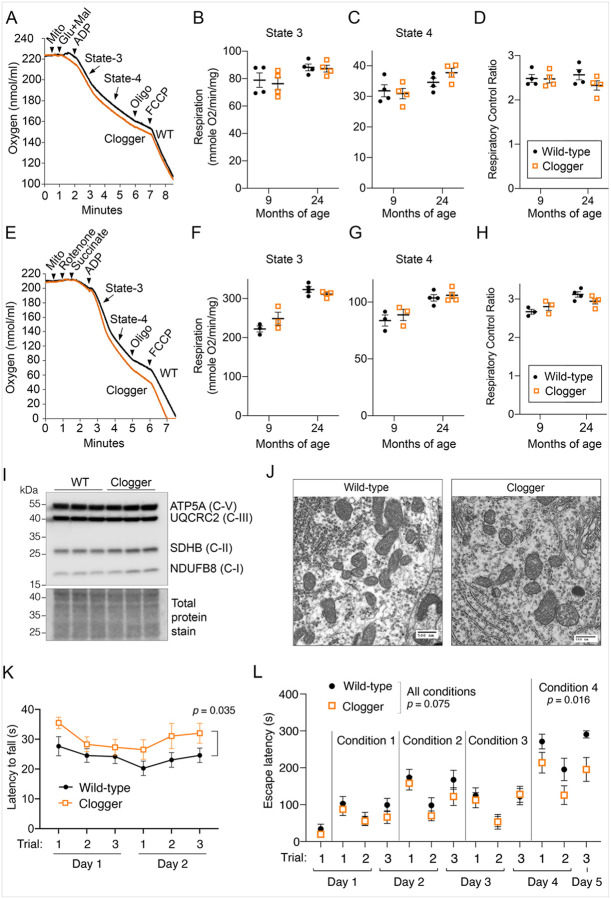
Mild mitochondrial protein import clogging with Ant1^p.A114P,A123D^ does not affect mitochondrial bioenergetics but moderately improves motor coordination and executive function. (A) – (D) Respirometry of purified brain mitochondria with complex I stimulated by glutamate (glu) and malate (mal). N = 4 mice/genotype/age group. Two measurements were taken per mouse, the average of which is plotted in (B)-(D). Data from different ages were analyzed separately due to a suspected batch effect. Independent repeated measures ANOVA within each age group (with measurement order as the within-subjects variable and genotype as the between-subjects variable) confirmed no effect of measurement order or genotype for any measure plotted in (B)-(D) or (F)-(H). FCCP, Trifluoromethoxy carbonylcyanide phenylhydrazone; Oligo, oligomycin. State 3 respiration is the maximal respiratory rate after addition of ADP. State 4 respiration is the respiratory rate after depletion of ADP. The respiratory control ratio is State 3 divided by State 4 respiratory rates. Decreased respiratory control ratio would be interpreted as increased mitochondrial damage. Horizontal bars indicate average values +/− SEM. (E) – (H) Respirometry of purified brain mitochondria with complex II stimulated by succinate and complex I inhibited by rotenone. N = 3 mice/genotype at 9 months of age and 4 mice/genotype at 24 months of age. Data collected and analyzed as in (A)-(D). (I) Immunoblot analysis of representative subunits of the respiratory complexes from purified brain mitochondria (9 months of age, each lane is an independent biological replicate). (J) Transmission electron microscopic analysis in the cell body of spinal cord ventral horn neurons showing no obvious changes to mitochondrial ultrastructure in non-paralytic clogger mice compared with wild-type (15 months of age). Scale bar is 500 nm. (K) Clogger mice perform better than wild-type on the accelerating rotarod at 19–22 months of age (n > 16 mice per genotype; n > 5 per sex per genotype). Average values are plotted +/− SEM. Data were analyzed with a two-way repeated measures ANOVA probing for an effect of sex and genotype, with Geisser-Greenhouse correction. Depicted is the main effect of genotype. (L) Clogger mice have moderately enhanced executive function, as judged by speed in removing obstacles from a 5 cm × 5 cm doorway to escape from a stressful environment. Mice were 20–23.5 months of age; n = 13 wild-type (5 females, 8 males), 15 cloggers (9 females, 6 males). Different “Conditions” are different obstacles. Average values are plotted +/− SEM. Data analyzed by repeated measures ANOVA with Geisser-Greenhouse correction.

The human ANT1^p.A114P^ protein is pathogenic, causing autosomal dominant Progressive External Ophthalmoplegia (adPEO)^19^. In addition to PEO and skeletal muscle phenotypes, ANT1-induced adPEO also associates with neurological and psychiatric phenotypes such as sensorineural hearing loss, cognitive impairment, dementia, bipolar and schizoaffective disorders^59–63^. ANT1^p.A123D^ is associated with myopathy and cardiomyopathy^20^. The Ant1^P.A114P,A123D^ /+ “clogger” mice were generated to facilitate phenotypic scoring and evaluate the *in vivo* effects of protein import clogging, as two mutations together resulted in TOM complex clogging that can directly be scored by biochemical assays^18^. To evaluate mitochondrial bioenergetics in the central nervous system of non-paralytic clogger mice, we measured oxygen consumption from purified brain mitochondria. Unlike skeletal muscle^18^, brain mitochondria from clogger mice at the age of 9 and 24 months do not have reduced maximal respiratory rate (state 3) when stimulating complex I (Figure 1A–B). ADP-depleted respiration (state 4) and the respiratory control ratio (state 3/state 4) were also unchanged (Figure 1C–D). These observations suggest that mitochondrial oxygen consumption is well-coupled with ATP synthesis and the maximal complex I-based respiratory capacity is not reduced in clogger brain mitochondria. Mitochondrial respiration was also unaffected when complex I is inhibited with rotenone and complex II-based respiration is stimulated with succinate (Figure 1E–H). Thus, mitochondrial respiration is unaffected or minimally affected in the brain of clogger mice. Consistent with this, we assessed the steady-state levels of respiratory chain proteins by immunoblot and found that representative subunits of complexes I, II, III and V were unaffected in clogger brain mitochondria (Figure 1I). We extended our analysis to the spinal cord by directly examining mitochondrial morphology using transmission electron microscopy. The data showed preserved mitochondrial morphology in ventral horn neurons in the non-paralytic clogger mouse spinal cord (15 months of age, Figure 1J). Taken together, these data suggest that mitochondrial structure and bioenergetic function are generally preserved in the central nervous system of clogger mice.

To explore the effects of mitochondrial protein import clogging on neurological function, we performed an array of behavioral assays on non-paralytic clogger mice. To ensure validity of the assays, we first assessed basic vision and motor function. Using optomotor response testing, we confirmed that visual acuity and contrast sensitivity are preserved in clogger mice at 17–18 months of age (Figure S1A-B). While 30-month-old clogger mice clearly exhibit skeletal muscle atrophy and weakness^18^, this effect appears to be age-dependent, as mice at the age of ~13 months have preserved muscle function, as suggested by treadmill exhaustion testing (Figure S1C-D) and swim speed (Figure S1E). We also tested ~20-month-old mice on an accelerating rotarod to measure motor coordination and balance. Surprisingly, we found that the clogger mice performed significantly better than wild-type mice (Figure 1K). This curious finding has previously been observed in another mild mouse model of mitochondrial disease^21^.

To assess general locomotor activity, we performed an open field test. When left undisturbed in a brightly lit open field for 10 minutes, female clogger mice move at a faster average speed compared with wildtype at 14–17 months of age (Figure S2A), reminiscent of another mouse model of mitochondria-induced Progressive External Ophthalmoplegia^22^. However, increased locomotor activity could not be attributed to increased anxiety-like behavior (Figure S2B-C). To assess cognitive function, we monitored animal behavior in several assays sensitive to short-term and long-term memory as well as executive function in this aged cohort (14–17 months of age). The Y-maze spontaneous alternation test relies on rodents’ natural tendency to explore new areas. When they repeatedly return to arms of the Y-maze that they most recently visited, this is interpreted as a defect in working spatial memory. Results from this assay suggested that clogger mice have preserved spatial working memory (Figure S2D). The novel object recognition test relies on rodents’ natural tendency to explore new objects after becoming familiarized with other objects over multiple prior training days. The data from this assay suggested that clogger mice have preserved long-term recognition memory (Figure S2E). The Morris water maze tests rodents’ ability to learn and remember the location of a hidden escape platform that they must swim to, which is unpleasant for rodents. Results from this assay suggested that clogger mice have preserved long-term spatial memory and learning (Figure S2F-H). Collectively, these data suggest that learning and both short-term and long-term memory are intact in clogger mice.

Finally, we tested executive function, which refers to higher-level processing used to control and coordinate behavior in response to changing task demands. To test this, we used a “puzzle box” assay in which mice are placed in a stressful environment and the amount of time it takes to escape through an obstructed exit doorway is used as a proxy for executive function^23,24^. We found that, for the most difficult obstruction (“Condition 4”), clogger mice were significantly better at escaping the stressful environment (Figure 1L). Therefore, clogger mice may have improved executive function in a manner that cannot be attributed to increased anxiety-like behavior. Overall, these behavioral data are critical for establishing that Ant1^p.A114P,A123D^ alone does not cause neurological dysfunction in non-paralytic mice. In fact, motor coordination and executive function appear to be equal or better than in wild-type mice.

### Transcriptional remodeling in the central nervous system.

**Figure 2:**
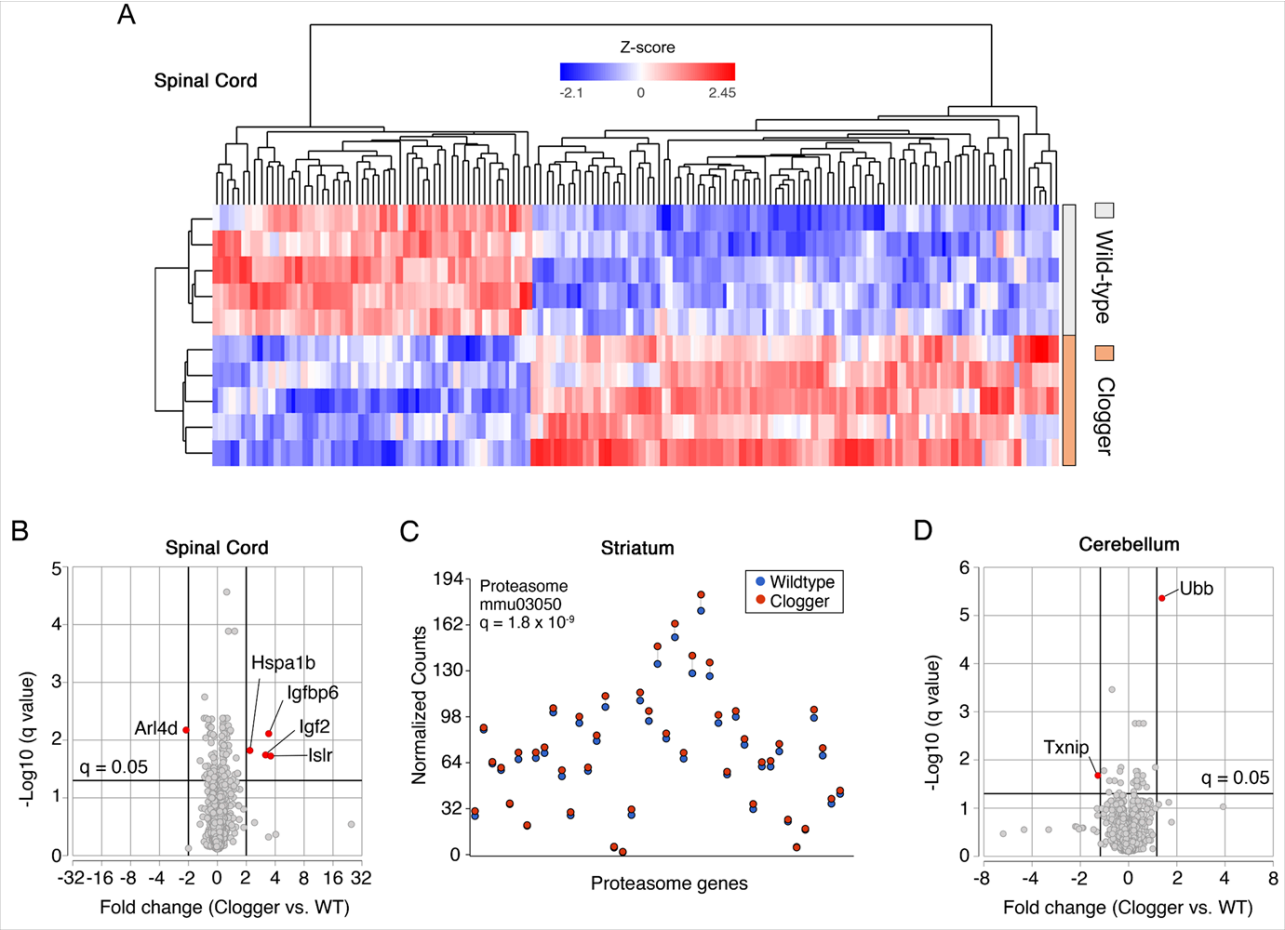
Transcriptomic analysis of neural tissues suggests cytosolic proteostatic stress in the clogger mice. (A) Heatmap of all genes that are differentially expressed in the spinal cord of clogger mice at 30 months of age, compared with wild-type (*q*<0.05). (B) Volcano plot of transcriptomics from the spinal cord at 30 months of age. (C) Partek Pathway analysis of transcriptomics data from the striatum at 24 months of age suggests global proteasome upregulation. (D) Volcano plot of transcriptomics from the cerebellum at 30 months of age shows significant upregulation of *Ubb*. Note that *Slc25a4* (*ANT1*) transcript level is consistently lower in the spinal cord (B) and cerebellum (D) from the Clogger mice due to partial instability and/or reduced transcription from the mutant *Ant1*^*p.A114P,A123D*^ allele (our unpublished observation).

To learn whether Ant1^p.A114P, A123D^-induced protein import clogging triggers specific stress responses, we first analyzed the spinal cord transcriptome because the spinal cord appears to be preferentially affected in clogger mice^18^. We found a robust transcriptional signature of 149 differentially expressed genes (*q* < 0.05) (Figure 2A, Supplementary Table 1). Among the four most upregulated genes were *Hspa1b*, *Igf2* and *Igfbp6* (Figure 2B). *Hspa1b* encodes one of the major stress inducible Hsp70 chaperones, which are important for protein folding and protection against proteostatic stress. Hsp70 is also involved in the stabilization and mitochondrial delivery of mitochondrial preproteins in the cytosol^25^. HSP70 genes are also transcriptionally activated by mitochondrial protein import clogging in human cells^9^ and yeast^18,26^. These observations suggest that transcriptional upregulation of HSP70 genes is a conserved response to mitochondrial protein import clogging.

Insulin-like growth factor-binding proteins (IGFBPs) are secreted proteins that bind insulin-like growth factors (IGFs) to regulate their transportation, localization, and function. IGFBP-6 is unique to this family in that it has a 20- to 100-fold higher affinity for IGF2 compared with IGF1^27^. Thus, co-induction of *Igfbp6* and *Igf2* in clogger-mouse spinal cords may be a coordinated stress response. Recent studies demonstrated that IGF2 can protect against neural damage through extracellular disposal of protein aggregates^28–30^.

We extended our RNA-Seq analysis to other regions within the central nervous system. In contrast to the spinal cord, we observed much more restricted transcriptional changes at the individual gene level in the cerebellum and striatum, with only 28 and 6 differentially expressed genes respectively (*q* < 0.05) (Figure S3A-3B, Supplementary Tables 2–3). Notably absent were any changes in genes involved in oxidative phosphorylation, consistent with a lack of mitochondrial respiratory defects. No genes involved in antioxidant defense were activated, which suggests the lack of oxidative stress. Instead, pathway analysis revealed global upregulation of proteasomal genes in the striatum (Figure 2C), which would be predicted if protein import clogging is causing mitochondrial Precursor Overaccumulation Stress (mPOS) in the cytosol^13,14,26,31^. Consistent with disturbed cytosolic proteostasis, we found that *Ubb* (ubiquitin B) is the most upregulated gene in the cerebellum (Figure 2D). *UBB* transcription was previously shown to be upregulated in neurons of Parkinson’s disease patients^32^. Related to the *Ubb* findings, we also noted significant increases in the *Dnajb11* transcript, which encodes an Hsp40 chaperone involved in unfolded protein responses and proteostasis (Supplementary Table 2). Finally, we point out that there was moderately but significantly increased expression of *Comt* transcript detected in the clogger cerebellum as well, which is a gene involved in the degradation of dopamine and norepinephrine that is commonly targeted by drugs used to treat patients in the early stages of PD. Overall, these transcriptional findings are consistent with disturbed extra-mitochondrial proteostasis by mitochondrial protein import clogging. Moreover, different regions of the central nervous system seem to have different stress responses. Consistent with the lack of detectable respiratory deficiency, the total number of mt-DNA encoded transcripts are not significantly changed in the neural tissues of the clogger mice when compared with the wild-type (Figure S3C).

### Protein import clogging worsens neurodegenerative phenotype in a mouse model of Parkinson’s disease.

We next used mouse genetics to test whether mild mitochondrial protein import stress with Ant1^p.A114P,A123D^ can worsen cytosolic protein aggregation and disease in an established mouse model of neurodegeneration. We crossed the clogger mice with hemizygous transgenic mice expressing the human a-synuclein with the A53T mutation, which is an autosomal dominant cause of familial Parkinson’s disease and a highly aggregation-prone protein^33^. Transgenic a-synuclein(A53T) mice (hereafter referred to as “a-syn” mice) develop overt neurological dysfunction at 12–16 months of age, which progresses to end-stage paralysis within 2–3 weeks of symptom onset^33^. Thus, to detect any additive effect of Ant1^p.A114P,A123D^ expression, we characterized the mice at 8–9 months of age (~20 mice/sex/genotype).

**Figure 3:**
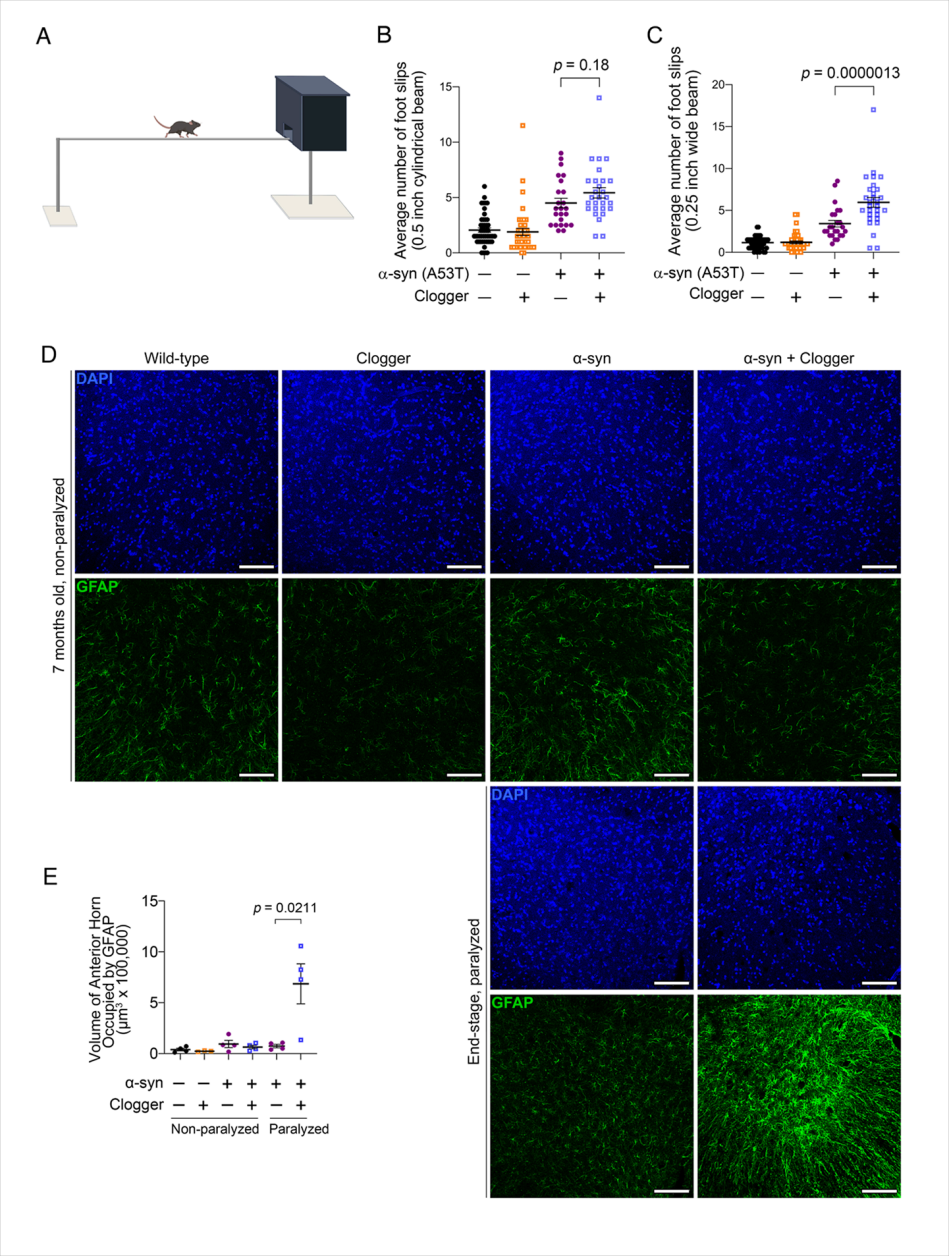
Mild protein import stress in the clogger mice impairs motor coordination and causes neuroinflammation only in the a-synuclein(A53T) transgenic background. (A) Schematic of the beam walking test. (B) Beam-walking test suggested marginally worsened coordination on a 0.5-inch cylindrical beam inclogger + a-Syn A53T double mutant compared with a-Syn A53T single mutant mice, as judged by the number of times each mouse’s hind limbs slipped off the beam. Data were first analyzed with a three-way ANOVA probing for effects of sex, a-synuclein, and clogger genotype. With no significant effect or interaction effect from sex, males and females were consolidated. Adjusted *p*-value that is shown is from a two-way ANOVA with Sidak’s multiple comparison’s test. Horizontal bars indicate average values +/− SEM (n=11–28 mice/sex/genotype, 8–9 months of age). (C) Beam-walking test suggested severely impaired coordination on a 0.25-inch rectangular beam in clogger + a-Syn A53T double mutant compared with a-Syn A53T single mutant mice. Data analyzed as in (B). There was a significant interaction between a-synuclein(A53T) and clogger genotypes (*p* = 7.5 × 10^−5^). Horizontal bars indicate average values +/− SEM (n=11–28 mice/sex/genotype, 8–9 months of age). (D) Representative images from immunofluorescence for the glial fibrillary acidic protein (GFAP) in the ventral horns of the lumbar spinal cord from control (Wild-type, Clogger) and end-stage a-syn mice with or without protein import clogging. Scale bar = 100 microns. (E) Increased volume occupied by GFAP in double mutant mice. Aggregate data for GFAP signal were generated exclusively from the ventral horns of five to eight sections per mouse at anatomically distinct lumbar spinal cord levels from three to four biological replicates per genotype. End-stage mice were 11.5–14.76 months of age. Non-paralyzed mice were 7 months of age +/− 2 weeks. *P* values were derived from student’s *t* test. Horizontal bars in (E) indicate average values +/−SEM.

Motor symptoms such as bradykinesia and postural instability are hallmarks of PD. We first assessed balance and coordination using the beam walking test. Mice were trained to walk across a thin beam and the number of times their paws slipped off the beam was scored as a measure of motor coordination (Figure 3A). As expected, a-syn mice slipped more often on both a 0.5-inch diameter cylindrical beam (*p* = 1.3 × 10^−5^), as well as a 0.25-inch rectangular beam (*p* = 7.2 × 10^−6^) (Figure 3B–C). Ant1^p.A114P,A123D^ expression significantly increased the number of foot slips on the smaller beam (*p* = 3.9 × 10^−6^), while showing a trend for further impairing performance on the larger beam. Importantly, Ant1^p.A114P,A123D^ expression had no effect in the wild-type background (Figure 3B–C). This suggests that mitochondrial protein import clogging and a-synuclein(A53T) expression act synergistically to impair motor function.

As another orthogonal approach to assess motor coordination, we measured the animals’ ability to avoid falling off an accelerating rotarod. In females, Ant1^p.A114P,A123D^ expression alone improved performance on the rotarod (Figure S4A), consistent with Figure 1K. This effect was abrogated by a-syn A53T expression. In males, a-syn (A53T) expression alone improved performance on the rotarod, consistent with previous reports^34^, but Ant1^p.A114P,A123D^ expression alone did not in this cohort. Increased performance in a-syn mice was abrogated by Ant1^p.A114P,A123D^ expression. Though complex, these effects are consistent with Ant1^p.A114P,A123D^ and a-syn A53T expression synergizing to impair motor coordination in mice. We followed these mice through end-stage paralysis to determine if Ant1^p.A114P,A123D^ expression reduced lifespan in a-syn mice (n=56–63 mice/genotype). We found no change in median lifespan in double mutant mice compared with a-syn alone (Figure S4B). Intriguingly, we noticed that in the longest-lived mice (upper quartile), Ant1^p.A114P,A123D^ expression significantly reduced maximum lifespan (log-rank *p* = 0.04 (Figure S4B), *t* test *p* = 0.028 (Figure S4C)). In sum, the data show that protein import clogging by Ant1^p.A114P,A123D^ can worsen motor deficits and modestly shorten maximum lifespan in a mouse model of a-synuclein(A53T)-induced PD.

We next assessed potential neuroanatomic etiologies of the observed phenotypes. Progressive degeneration of dopaminergic neurons in the substantia nigra, pars compacta (SNc) are a hallmark of PD. We did not observe a reduction in TH-positive neurons in the SNc of presymptomatic a-syn mice compared with the wild type controls (Figure S5), consistent with previous reports in this mouse model^33^. The number of TH-positive neurons was slightly increased in the SNc of a-syn + Ant1^p.A114P,A123D^ compared with the a-syn single mutant mice at the end stage. Dopaminergic neurons of the SNc project to the striatum in part to control motor function. We did not observe a significant difference in TH-intensity in the striatum between a-syn and a-syn + Ant1^p.A114P,A123D^ mice (Fig. S6). Additionally, we did not observe significant differences in cell death in the SNc as judged by TUNEL assay (Fig. S7). Ubiquitin positive inclusion bodies are present in the midbrain regions in this particular mouse model overexpressing human a-syn A53T^33^. We therefore assessed for the formation of ubiquitin inclusion bodies within the midbrain regions, to see whether clogging further challenges cytosolic proteostasis and promotes inclusion formation in the a-syn background. Within the midbrain reticular nucleus and the superior colliculus (two regions responsible for the initiation of movement) we observed a mild but insignificant trend towards increased inclusion body formation in a-syn + Ant1^p.A114P,A123D^ mice when compared with single mutant a-syn mice (Fig. S8).

Next, we turned to spinal cord motor neurons as they are among the earliest cells affected in a-syn A53T mice^35^. In end-stage a-syn mice, we observed a significant decrease in the number of vesicular acetylcholine transporter (VAChT)-positive motor neurons in the lumbar spinal cord, which was minimally affected by clogger expression (Figure S9A-B). Nissl staining of end-stage mice showed a trend towards reduced number of anterior horn neurons and increased number of chromatolytic neurons in the double mutant mice (Figure S9C-E). Consistent with increased chromatolysis, the stromal size of the anterior horn neurons in the end stage double mutant were significantly larger than that of the a-syn mice (Figure S9F)^36^. Importantly, we observed a striking increase in neuroinflammation in the end-stage double mutant mice compared with a-syn only, as judged by GFAP staining (Figure 3D–E). Taken together, these data support increased neuronal damage in the spinal cord of end-stage double mutant mice compared with a-syn only.

Does protein import clogging aggravate additional phenotypes of a-syn mice? Up to 40% of PD patients experience anxiety^37^. We found that mutant a-syn expression significantly increased anxiety-like behavior in the open field test (*p* = 1.3 × 10^−4^) (Figure S10A). While in the center zone, double mutant mice remained closest to the edge zone on average, suggesting Ant1^p.A114P,A123D^ expression moderately increases anxiety-like behavior in a-syn, but not wild-type mice (Figure S10B). Spontaneous locomotor activity was increased in a-syn mice (*p* < 10^−15^), as previously reported^38^, which was not affected by Ant1^p.A114P,A123D^ (Figure S10C). In the novel object recognition test, a-syn expression caused a significant defect in long-term object memory (*p* = 2 × 10^−6^), with no effect from Ant1^p.A114P,A123D^ expression (Figure S10D). The total time spent exploring either object was largely unaffected by genotype (Figure S10E). In the Y-maze spontaneous alternation test, a-syn expression caused a significant defect in short term spatial memory (*p* = 2.5 × 10^−5^), with no effect of Ant1^p.A114P,A123D^ expression (Figure S10F). The total number of arm entries in the Y-maze was increased by a-syn expression (*p* = 1.6 × 10^−4^), with no effect of Ant1^p.A114P,A123D^ expression (Figure S10G). It is likely that the memory defects in a-syn mice are already severe by 9 months old, precluding detection of any potential additive effect from Ant1^p.A114P,A123D^ expression.

### Ant1^p.A114P, A123D^ does not affect mitochondrial bioenergetics in a-syn mice.

**Figure 4:**
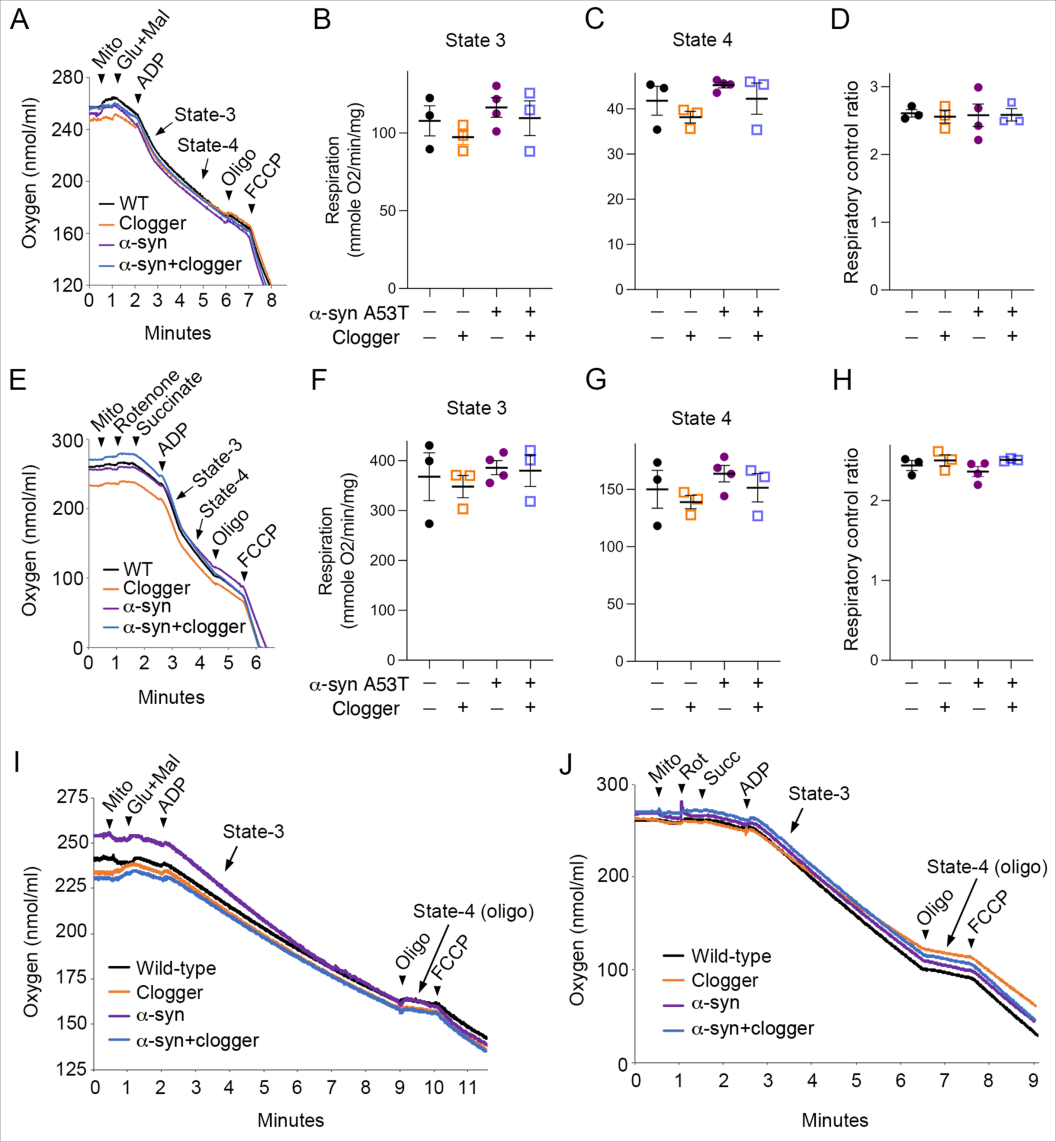
There is no detectable respiratory deficit in 9-month-old a-syn or a-syn plus clogger double mutant mice. (A) – (D) Respirometry of purified brain mitochondria with complex I stimulated by glutamate (glu) and malate (mal). Two measurements were taken per mouse, the average of which is shown as a data point in (B)-(D). FCCP, Trifluoromethoxy carbonylcyanide phenylhydrazone; Oligo, oligomycin; Glu, glutamate; Mal, malate. State 3 respiration is the maximal respiratory rate after addition of ADP. State 4 respiration is the respiratory rate after depletion of ADP. The respiratory control ratio is State 3 divided by State 4 respiratory rates. Decreased respiratory control ratio would be interpreted as increased mitochondrial damage. Two measurements were performed per mouse. Each dot represents the average value from each mouse (n=3–4/mice/genotype, 9 months of age +/− 2 weeks). (A) – (H) Respirometry of purified brain mitochondria with complex II stimulated by succinate (glu) and complex I inhibited by rotenone. Each dot represents the average value from each mouse (n=3–4 mice/genotype, 9 months of age +/− 2 weeks). Horizontal bars indicate average values +/− SEM. (I) Respirometry from synaptosomal fractions with complex I stimulated as above. (J) Respirometry from synaptosomal fractions with complex II stimulated as above.

There are substantial data to suggest that a-synuclein and other protein aggregates can impact mitochondrial bioenergetics, while other studies failed to generate consistent results^5^. We tested the possibility that a synergistic defect in mitochondrial respiratory function may contribute to the enhanced double mutant phenotypes. We used 9-month-old mice asymptomatic mice, as this may avoid the onset of large neural tissue lesions in end-stage mice that may indirectly affect mitochondrial function and confound the interpretation of the data. We again measured complex I- and complex II-stimulated respiration from purified brain mitochondria. We found that a-synuclein(A53T) expression did not reduce respiratory rates. More importantly, no respiratory deficiency was observed even in the Ant1^p.A114P,A123D^ + a-syn double mutant mice (Figure 4A–H). Because Percoll-purified mitochondria are mostly non-synaptosomal, and a-syn primarily localizes to pre-synaptic sites, we also tested respiration from the synaptosomal brain fractions. These fractions are impure, containing microsomal membranes and myelin. Respirometry from synaptosomal fractions suggested that wild-type, a-syn (A53T), and double mutant mice all have similar respiratory rates (Figure 4I–J). To assess the possibility that regional differences in mitochondrial function were masked in the whole brain bioenergetics experiments, we assessed COX activity and respiratory chain protein levels from crude mitochondrial fractions isolated from various brain regions and the spinal cord. The data did not detect changes in COX activity or respiratory chain protein levels at 9 months of age (Figures S11 & S12). This contrasts with previous work showing that Cytochrome *c* oxidase (COX) activity was reduced in the spinal cord of end-stage, paralyzed a-syn mice^39^. We note that our bioenergetic experiments were performed at 9 months of age, the age at which we observe motor defects but well before paralysis onset. Taken together, our data strongly suggest that bioenergetic defects do not underlie the synergistic effect between mitochondrial protein import clogging and a-syn proteotoxicity that aggravates motor deficits and neurodegenerative phenotype.

### Mitochondrial protein import clogging increases the size of phosphorylated a-synuclein aggregates.

We hypothesized that mitochondrial protein import clogging would cause mPOS and increase proteostatic burden in the cytosol, which may in turn affect a-synuclein aggregation. To narrow our focus to a particular region of the central nervous system, we performed detergent solubility studies to assess where a-synuclein(A53T) is preferentially detergent-insoluble, which we use as a proxy for aggregation. We probed the Triton-insoluble fractions from the forebrain, midbrain, cerebellum and spinal cords of end-stage a-syn mice for a-synuclein phosphorylated at serine 129 (P-a-syn). P-a-syn accumulates in pathological aggregates and is a more specific marker for protein aggregation and disease activity compared with non-phosphorylated a-synuclein^40^. We found that insoluble P-a-synuclein preferentially accumulates in the spinal cord (Figure S13A), consistent with previous studies^33^. We therefore focused our biochemical studies of protein aggregation on the spinal cord.

**Figure 5:**
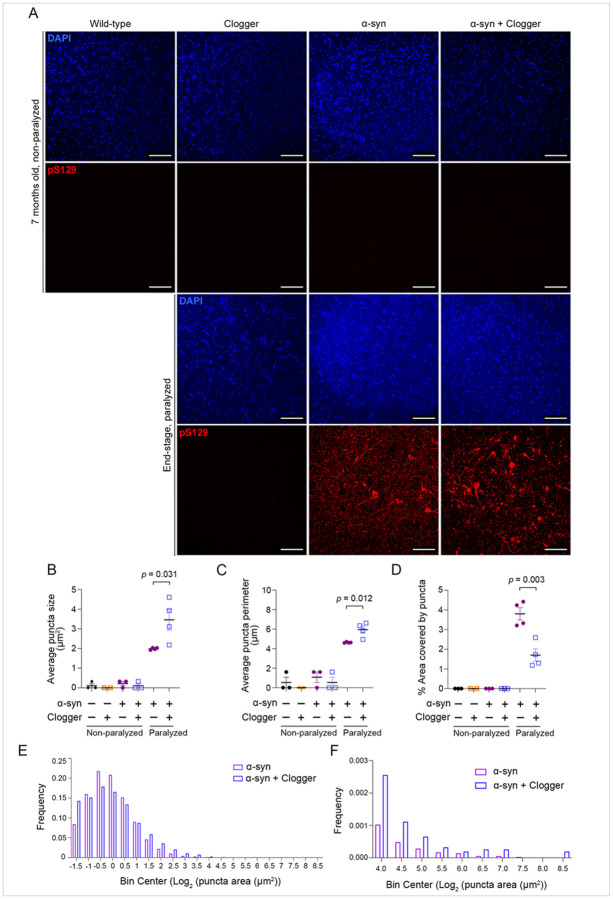
Increased size of a-syn phospho-S129 puncta in double mutant mice. (A) Representative images from immunofluorescence for a-synuclein phosphorylated at S129 in the ventral horns of the lumbar spinal cord from control (Wild-type, Clogger) and end-stage a-syn mice with or without protein import clogging. Scale bar = 100 microns. (B) Increased average P-a-syn puncta size in double mutant mice. (C) Increased average P-a-syn puncta perimeter in double mutant mice. (D) Reduced % area covered by P-a-syn puncta in double mutant mice. (E) Frequency distribution of P-a-syn puncta size. (F) Frequency distribution as in (E), but focused on larger puncta that are not visible in (E). Aggregate data were generated exclusively from the ventral horns of three sections per mouse at anatomically distinct lumbar spinal cord levels from two to four biological replicates per genotype. End-stage mice were 12–14.75 months of age. Non-paralyzed mice were 7 months of age +/− 2 weeks. *P* values were derived from student’s *t* test. Horizontal bars in (B-D) indicate average values +/−SEM.

First, immunoblot analysis showed that P-a-syn preferentially forms higher molecular weight species in end-stage double mutant compared with a-syn only mice (Figure S13B-C), likely representing increased oligomerization. To quantitatively assess the size and number of a-synuclein aggregates, we performed immunofluorescence on the lumbar spinal cord. At 7 months of age, neither a-syn nor double mutant mice showed significant pathology when probing for P-a-syn (Figure 5A). In end-stage mice, we found many puncta staining positive for P-a-syn, suggesting widespread protein aggregation as expected (Figure 5A). We found that the average size of P-a-syn puncta was increased in the ventral horns of double mutant mice compared with a-syn only (Figure 5B, E and F). Consistent with increased puncta size, the average perimeter of puncta was also increased in double mutants (Figure 5C). We also observed a decrease in the percentage of area occupied by P-a-syn in the double mutants (Figure 5D), suggesting altered distribution of P-a-syn in ventral motor horn of the double versus single mutant mice. These data support an overall increased propensity of a-syn aggregation in the cell body of double mutant relative to a-syn single mutant mice.

### Protein import clogging increases the aggregation of mitochondrial proteins in the a-synuclein background.

If cytosolic proteostatic stress from un-imported mitochondrial preproteins is increasing a-syn aggregation, then we would expect a reciprocal increase of aggregation of mitochondrial proteins in the presence of a-synuclein. We used detergent solubility studies to test this hypothesis in end-stage mouse spinal cords, reasoning that the pool of detergent-insoluble mitochondrial proteins should increase in the double mutants compared with a-syn alone. Indeed, we found that mitochondrial proteins such as SDHA, SDHB, VDAC and TIMM23 are significantly enriched in the Triton-insoluble spinal cord fractions from a-syn + clogger relative to the a-syn mice (Figure S14A-F). The presence of proteins such as SDHA in the soluble fraction is accordingly reduced in the a-syn plus clogger relative to the a-syn mice. For some mitochondrial proteins (e.g., ATP5A and NDUFB8), there was a mild increase in their levels in the insoluble fractions that was not statistically insignificant (Figure S14A-B). On the other hand, several mitochondrial matrix proteins (MDH2 and HSP60) are undetectable in the Triton-insoluble fractions in a-syn and a-syn plus clogger mice (Figure S14E). Based on these observations, we conclude that aggregation of unimported mitochondrial proteins is likely dependent on their individual biochemical properties with membrane proteins more susceptible to aggregation.

**Figure 6:**
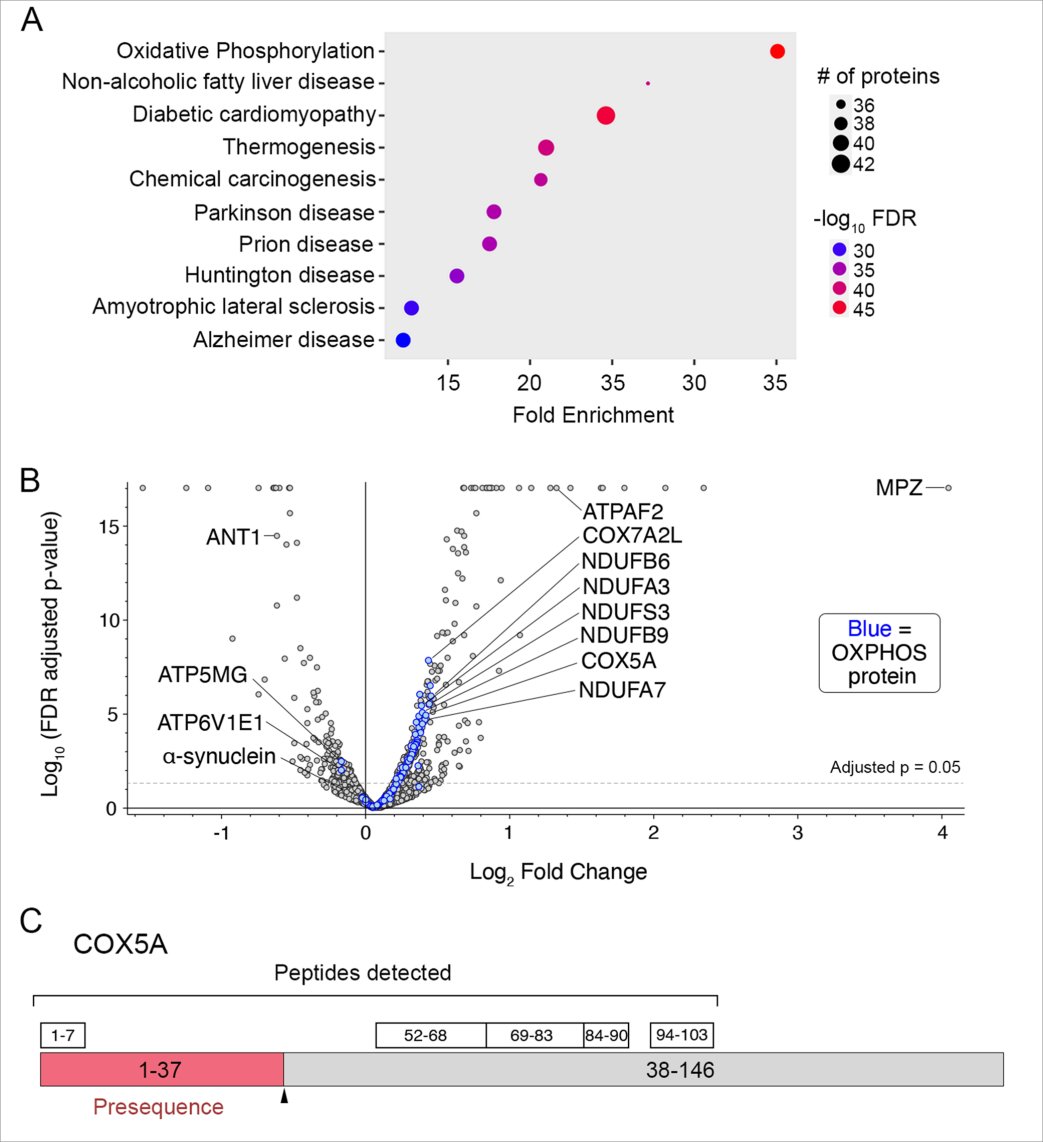
Protein import clogging increases aggregation of mitochondrial proteins in the a-synuclein background *in vivo*. (A) KEGG pathway analysis of the proteins significantly increased (FDR adjusted *p*< 0.05) in the insoluble fraction of double mutant end-stage mouse spinal cord compared with a-syn alone (9–13.4 months of age), as determined by TMT-based quantitative proteomics. FDR, false discovery rate. (B) Volcano plot of TMT proteomics experiment of Triton-insoluble proteins with OXPHOS proteins labeled as blue dots. (C) Schematic of COX5A protein and the five peptides quantified in the TMT proteomics experiment, shown above the protein (38% coverage). In red is the COX5A pre-sequence (residues 1–37) that is normally cleaved and degraded if the protein is efficiently targeted into mitochondria.

To gain a global, unbiased view of protein aggregation, we performed tandem mass tag (TMT) quantitative proteomics on the detergent insoluble fractions from end-stage double mutant versus a-syn mouse spinal cords (9–13.3 months of age). We found 185 proteins significantly increased in the double mutants (FDR-adjusted *p* < 0.05, Supplementary Table 4). Of these, 55 were ascribed to the “mitochondrion” Cell Component, which was significantly overrepresented (GO:0005739, FDR < 10^−15^). KEGG pathway analysis showed “oxidative phosphorylation” as the most enriched group of proteins that are increased in the insoluble fractions from double mutant spinal cords (Figure 6A–B, FDR < 10^−42^). This was not driven by increased total a-syn levels (Figure 6B). Interestingly, multiple neurodegenerative disease KEGG pathways were also enriched, most notably PD (Figure 6A). Finally, we detected a unique peptide within mitochondrial targeting presequence of COX5A, a protein significantly increased in the double mutant aggregates (Figure 6C). As this peptide is cleaved and degraded if COX5A efficiently enters mitochondria, this observation suggests the presence of un-imported mitochondrial preproteins within the protein aggregates. The enrichment of mitochondrial proteins appears to be specific for the insoluble fraction, as TMT proteomics of the soluble fractions did not show enrichment of mitochondrial proteins (Figure S14G).

Taken together, the data suggest that mitochondrial protein import clogging may challenge cytosolic proteostasis by causing un-imported mitochondrial preproteins to accumulate and aggregate in the cytosol with or without co-aggregation with a-synuclein.

## Discussion

Two important hallmarks shared among prevalent neurodegenerative diseases such as PD, Lewy Body Dementia, Alzheimer’s disease, and ALS are mitochondrial dysfunction and cytosolic protein misfolding/aggregation. Whether and how these hallmarks interact in disease pathogenesis is an open question. Studies in yeast and cultured human cells over the last decade have shown that a diverse range of mitochondrial insults can affect protein import and cause the toxic accumulation and aggregation of mitochondrial preproteins in the cytosol, a process termed mPOS^13,14^. This raises the question as to whether mPOS can contribute to or modify the course of disease pathology *in vivo* by affecting cytosolic proteostasis independent of bioenergetic defects of mitochondria. The critical barrier to addressing this question has been modeling protein import defects *in vivo* without confounding oxidative phosphorylation deficiency and/or lethal shutdown of mitochondrial biogenesis. In this study, we overcome this roadblock by establishing that mild protein import clogging by Ant1^p.A114P,A123D^ has little to no effect on bioenergetics in the central nervous system of heterozygous mice. As a first step to assessing mPOS in neural damage, we induced mitochondrial protein import clogging by expressing Ant1^p.A114P,A123D^ in an established mouse model of proteostatic stress caused by expression of human a-synuclein with a pathogenic A53T mutation. The mutant a-syn is a highly aggregation-prone protein that causes an autosomal dominant cause of familial PD^33^. We tested whether mitochondrial protein import clogging and mPOS alter the proteostatic dynamics and pathogenicity of a-synuclein independent of mitochondrial bioenergetics.

The data presented provide foundational evidence that mildly reduced mitochondrial protein import efficiency is sufficient to accentuate motor coordination defects and a-synuclein aggregation independent of bioenergetics. Mild import stress likely increases a-synuclein aggregation by challenging the global proteostatic network in the cytosol, potentially causing co-aggregation of a-synuclein with un-imported mitochondrial preproteins. These biochemical changes correlate with increased inflammation and neuronal chromatolysis in the spinal cord where reside the earliest cells affected in a-syn A53T mice relative to the midbrain^35^. Importantly, bioenergetic defects cannot explain the phenotype augmentation by Ant1^p.A114P,A123D^ in a-syn mice. We did not observe a reduction in mitochondrial respiration, cytochrome *c* oxidase activity, or loss of electron transport chain stability in the central nervous system of double mutant mice. This may seem contrary to the observation that OXPHOS proteins appear to be aggregation prone in the double mutants. It is likely that mild depletion of specific proteins/complexes does not cause significant deficiency in OXPHOS, as previously proposed^41^. Taken together, although we cannot completely exclude a subtle effect from undetectable bioenergetic deficiency, the data suggest that the cytosolic accumulation of un-imported mitochondrial preproteins (i.e. mPOS) can contribute to neural damage. Our finding therefore adds a new layer to the complex relationship between mitochondrial dysfunction and neuropathogenic protein aggregates. We propose that this finding could have broad pathophysiologic and therapeutic implications, and warrants testing of mPOS in further PD mouse models and other models of neurodegeneration such as Alzheimer’s and ALS in which mitochondrial abnormalities have been extensively reported.

We chose to test mPOS in a model of PD because it has long been appreciated that mitochondrial dysfunction can cause cytosolic a-synuclein aggregation, but the molecular mechanisms have not yet been elucidated. Moreover, the roles of bioenergetic deficiency and/or non-bioenergetic mechanisms remains unclear. In line with a role for bioenergetic deficiency, toxins that inhibit mitochondrial complex I, such as MPTP and rotenone, cause a-synuclein aggregation and PD in humans, human cells, and animal models^42–47^. Mutations in mtDNA in dopaminergic neurons, which would certainly cause bioenergetic deficiency, can induce the formation of cytosolic inclusions that contain mitochondrial proteins^48,49^. mtDNA mutations have also been shown to cause degeneration of dopaminergic neurons and motor deficit when synergized with the loss of Parkin, an E3 ubiquitin ligase mutated in an early onset form of PD^50^. These findings implicate mitochondrial bioenergetics in pathogenic a-synuclein aggregation. We previously speculated that bioenergetic deficiency itself may lead to reduced protein import and mPOS^14^. For example, inhibition of complex I with MPTP reduces mitochondrial protein import in cells as well as *in vitro*^51^, and reduced protein import aggravates seeding of a-synuclein aggregates in cell models^52^. Moreover, mitochondrial proteins are found in a-synuclein aggregates in PD patient brains^53–56^. Overall, bioenergetic deficiency may indeed play a role in PD pathophysiology with or without mPOS. Complementary to this, we show proof-of-concept that mPOS can be an additional, non-bioenergetic pathogenic consequence of mitochondrial dysfunction in neural damage. Additional studies are needed to further dissect the interplay of bioenergetic-dependent and - independent factors in PD.

Our finding that mild mitochondrial protein import defect is sufficient to alter the course of a-synuclein induced neural damage could have important implications for better understanding of PD. As mentioned above, mitochondrial stress has been widely reported in PD patient samples. It is expected that genetic and environmental PD inducers (e.g., defective mitochondrial protein quality control and exposure to MPTP or rotenone) would reduce proton pumping across the IMM, which results in reduced membrane potential and protein import^14^. On top of this, a-synuclein itself may impair mitochondrial protein import by directly binding to the TOM20 import receptor^57^, and/or by indirectly decreasing mitochondrial protein import through an effect on other mitochondrial processes such as membrane potential maintenance^58^, electron transport chain integrity, mitochondrial dynamics/transport and quality control^59^. As expression and accumulation of a-synuclein increases in PD-affected neurons with normal aging^60^, it is possible that an age-dependent increase in a-synuclein level gradually reduces mitochondrial protein import, thereby causing mPOS to aggravate its own aggregation in the cytosol. A similar process may happen in certain forms of familial PD. Notably, loss of PINK1, which is a recessive cause of PD, has also been convincingly shown to reduce mitochondrial protein import^61^.

In summary, we utilized our unique clogger mouse model to uncover the neurological effects specific to mitochondrial protein import stress and subsequent mPOS. We found that mild import stress can readily be mitigated in healthy mice but enhances protein aggregation and motor deficits in a mouse model of PD without a detectable effect on mitochondrial bioenergetics. Thus, mitochondrial protein import efficiency and mPOS may be disease modifying factors in neurodegeneration independent of OXPHOS.

## Methods

### Mouse studies

All procedures were approved by the Animal Care and Use Committee (IACUC) at State University of New York Upstate Medical University and were in accordance with guidelines established by the National Institutes of Health. *Slc25a4* ^p.A114P,A123D^/+ mice, or “clogger” mice, were generate as previously described^18^. After >10 back crosses with the C57BL/6NTac females (Taconic Catalog no: B6-F), male *Slc25a4* ^p.A114P,A123D^/+ mice were crossed with female C57BL/6 mice with transgenic expression of the human *SNCA* gene (coding for the a-synuclein protein) harboring the pathogenic A53T mutation (Jackson Lab #006823)^33^. *SNCA* expression is driven by the mouse prion promoter in these mice. After the first cross between clogger and *SNCA* transgenic mice, double mutant mice were backcrossed against female C57BL/6NTac mice. Where a-syn mice were tested, all wild-type and clogger control mice were littermates.

Animals in the a-syn background were examined daily for signs of illness for determination of “end stage” for lifespan, histological and biochemical analysis. An animal was considered “end stage” once it displayed paralysis and was so severely moribund that it was determined unlikely to survive more than an additional 48 hours, as judged by an experienced technician in our Department of Laboratory Animal Resources. A mouse was considered severely moribund if it also exhibited the following clinical signs: inability to eat, drink or eliminate; severe dehydration; labored breathing; severe lethargy. Other parameters used that were used in lifespan determination, but not specific to “end stage” a-syn mice were edema, sizable abdominal enlargement or ascites, significant skin lesions exposing muscle, progressive dermatitis, or a severely ulcerated or bleeding tumor.

### Mitochondrial isolation and purification

For mitochondrial respiratory studies, mice were sacrificed by decapitation without CO_2_ asphyxiation or anesthetic. Neural tissues were rapidly dissected (<60 seconds) and placed in 1 mL of ice-cold Isolation Medium (IM) (225 mM Mannitol, 75 mM sucrose, 5 mM HEPES-KOH pH 7.4, 1 mM EGTA, 0.1% BSA). Quick dissection of the spinal cord was enabled via hydraulic extrusion with ice-cold PBS^62^. Tissue was minced on ice while submerged in IM as soon as possible. For crude mitochondrial fraction isolation (i.e. for cerebellum, spinal cord, and brain stem), well-minced tissue was homogenized with 4 strokes by hand in a 2 mL glass dounce homogenizer (pestle B, clearance 0.0005–0.0025 inches), followed by differential centrifugation at 4°C. First, homogenate was centrifuged at 2,000g for 5 minutes. Supernatant was then centrifuged at ~21,000g for 20 minutes. Pellet was resuspended in 0.5 mL IM and centrifuged again at ~21,000g for 20 minutes. Again, pellet was resuspended in 0.5 mL IM and centrifuged again at ~21,000g for 20 minutes. The final pellet was resuspended in 0.2 mL IM.

Mitochondria were purified from the forebrain, which in our studies includes all neural tissue anterior to the junction between the cerebral cortex and the cerebellum. This was done using discontinuous Percoll gradient centrifugation, essentially as described^63^. Minced forebrains were homogenized with three strokes with the pestle at 13,900 rpm in ice cold 7 mL IM. Homogenate was then centrifuged for 5 minutes at 2,000g. Resulting supernatant was centrifuged at 13,000 g for 12 minutes. Pellet was then resuspended in 2 mL IM containing 12% Percoll and used to build a discontinuous gradient using the following Percoll concentrations in isotonic IM: 5 mL 40%, 5 mL 23%, and ~2.5 mL 12% (mito). Gradients were ultra-centrifuged in a swinging-bucket rotor (SW 41) at 18,500 rpm (~43,000 g) for 20 minutes with slow acceleration and deceleration. Purified non-synaptosomal mitochondria were retrieved from the 23/40% interface, and synaptosomes were collected from the 23/12% interface. Both fractions were then diluted with ~25 mL IM without Percoll, and centrifuged at 13,000g for 15 minutes. Mitochondrial pellet was resuspended in 1 mL IM and centrifuged at 13,000g for 12 minutes. Mitochondria were then washed two more times in 1 mL ice-cold IM, and finally resuspended in 80 ml for non-synpatosomal pure mitochondria, and 200 ml for synaptosomes. Protein concentration was then determined by Bradford assay.

### Mitochondrial oxygen consumption measurements

Oxygen consumption assays were performed using Oxygraph system form Hansatech, which has a small water-jacketed (37°C), magnetically stirred chamber sitting atop a Clark electrode. Each reaction occurred in 0.5 ml Respiratory buffer (125 mM KCl, 4 mM K_2_HPO_4_, 3 mM MgCl_2_, 1 mM EGTA, 20 mM HEPES-KOH pH 7.2). 150 mg or 300 mg of non-synaptosomal and synaptosomal mitochondria, respectively, were added first. Then, for Complex I-based respiration, reagents were added in the following sequence at the indicated final concentrations: Glutamate (5 mM) + Malate (2.5 mM); ADP (200 mM); oligomycin (3 mg/ml); FCCP (0.2 mM). For Complex II: rotenone (5 mM); succinate (10mM); ADP (300 mM); oligomycin (3 mg/ml); FCCP (0.2 mM).

### Respiratory complex activity assay

COX activity assay was performed essentially as described^64^. Oxidized cytochrome c (Sigma-Aldrich: C2506) was reduced with sodium dithionate and dialyzed overnight in potassium phosphate buffer, followed by spectrophotometric confirmation that Cytochrome c was reduced (OD 550/560 ratio > 6). For the activity assay, snap-frozen crude spinal cord mitochondria were thawed on ice and 1 mL reaction tube was prepared with 20 mM potassium phosphate, pH 7.0, 0.45 mM DDM and 15 mM reduced cytochrome C. At time zero, 0.02 mg mitochondria (spinal cord) or 0.2 mg (brainstem, forebrain, cerebellum) were added to the reaction and OD550 was monitored every 2 seconds for 5 minutes. The slope over the first 12 seconds of the assay was used to determine the rate constants.

### Electron microscopy

For electron microscopy, *Slc25a4* ^p.A114P,A123D^/+ mice and littermate controls were processed as previously described^65^. Briefly, mice were anesthetized with isoflurane and perfused intracardially with PBS initially, followed by fixative (1% paraformaldehyde, 1% glutaraldehyde, 0.12 M sodium cacodylate buffer pH 7.1, and 1 mM CaCl_2_). Perfused animals were refrigerated overnight, and CNS tissues dissected the next day and processed for TEM. The samples were examined with a JOEL JEM1400 transmission electron microscope and images were acquired with a Gaten DAT-832 Orius camera.

### Detergent solubility studies

Triton Lysis Buffer (0.5% Triton X-100, 150 mM NaCl, 50 mM HEPES-KOH pH 7.4, 1 mM EDTA with protease and phosphatase inhibitors (Roche)) was added to dissected and snap-frozen tissues at a ratio of 6 μL/mg of tissue. Tissue was minced and homogenized in a 2 mL dounce homogenizer, then incubated on ice for 30 minutes. Triton-insoluble fractions were collected by centrifugation at 21,000g for 20 minutes, then washed twice by vortexing in Triton Lysis Buffer. Soluble supernatant was centrifuged an additional two times at 21,000g for 30 minutes each. Triton insoluble fractions were solubilized with 5% SDS and 8M Urea, mixed with pipetting and sonicated for ~10 seconds. Samples were then incubated at 42°C for 30 minutes before centrifugation at 16,000g for 20 minutes at room temperature. Supernatant was the final “Triton insoluble fraction” and was either directly loaded onto an SDS-PAGE gel for Western blot analysis or processed for quantitative mass spectrometry as described below.

### Sample processing for Quantitative Mass Spectrometry

Five biological replicates of a-syn and double mutant samples were prepared for multiplexed quantitative mass spectrometry as above. Samples were buffer exchanged on a 3 kDa molecular weight cutoff filter (Amicon 3k Ultracel) using 4 additions of 10 mM triethylammonium bicarbonate, pH 8.0 (Thermo). One hundred μg was taken for digestion using an EasyPep Mini MS sample prep kit (Thermo, A40006). To each buffer-exchanged sample, 65 μL of lysis buffer was added followed by 50 μL of reduction solution and 50 μL of alkylating solution. Samples were incubated at 95°C for 10 minutes, then cooled to room temperature. To each sample 5 μg of trypsin / Lys-C protease was added and the reaction was incubated at 37°C overnight. Half of each digest was used for subsequent labeling. TMT reagents were reconstituted with 40 μL acetonitrile (ACN) and the contents of each label added to a digested sample. After 60 min, 50 μL of quenching solution was added, consisting of 20% formic acid and 5% hydroxylamine (v/v) in water. The labeled digests were cleaned up by a solid-phase extraction device contained in the EasyPep kit, and dried by speed-vac. The individually labeled samples were dissolved in in 50 μL of 30% ACN and 0.2% trifluoroacetic acid (v/v) in water, and 15 μL of each was used to create a pooled sample consisting of 150 μg.

### Fractionation

Following an LC-MS experiment to check digestion and labeling quality of the pooled samples, 100 μg of the pooled sample was fractionated using a Pierce High pH Reversed-Phase Peptide Fractionation Kit (part # 84868), per the manufacturer’s instructions for TMT-labeled peptides. In brief, samples were dissolved in 300 μL of 0.1% trifluoroacetic acid in water and applied to the conditioned resin. Samples were washed first with water and then with 300 μL of 5% ACN, 0.1% triethylamine (TEA) in water. The second wash was collected for analysis. Peptides were step eluted from the resin using 300 μL of solvent consisting of 5 to 50% ACN with 0.1% TEA in eight steps. All collected fractions were dried in a speed-vac.

### LC-MS/MS

Dried fractions were reconstituted in 25 μL of load solvent consisting of 3% ACN and 0.5% formic acid in water, and a 5 μL aliquot was diluted 1:3 with the same solvent. Of these 15μL, 2 μL were injected onto a pulled tip nano-LC column (New Objective, FS360-75-10-N) with 100 μm inner diameter packed to 32 cm with 1.9 μm, 100 Å, C18AQ particles (Magic 2, Premier LCMS). The column was maintained at 50°C with a column oven (Sonation GmbH, PRSO-V2). The peptides were separated using a 135-minute gradient consisting of 3 – 12.5% ACN over 60 min, 12.5 – 28% over 60 min, 28 – 85 % ACN over 7 min, a 3 min hold, and 5 min re-equilibration at 3% ACN. The column was connected inline with an Orbitrap Lumos (Thermo) via a nanoelectrospray source operating at 2.3 kV. The mass spectrometer was operated in data-dependent top speed mode with a cycle time of 3s. MS^1^ scans were collected from 375 – 1500 m/z at 120,000 resolution and a maximum injection time of 50 ms. HCD fragmentation at 40% collision energy was used followed by MS^2^ scans in the Orbitrap at 50,000 resolution with a 105 ms maximum injection time.

### Database searching and reporter ion-based quantification

The MS data was searched using SequestHT in Proteome Discoverer (version 2.4, Thermo Scientific) against the *M. Musculus* proteome from Uniprot, containing 50961 sequences and a list of common laboratory contaminant proteins. Enzyme specificity for fully tryptic with up to 2 missed cleavages. Precursor and product ion mass tolerances were 10 ppm and 0.02 Da, respectively. Cysteine carbamidomethylation, TMT 10-plex at any N-terminus and TMT 10-plex at lysine were set as a fixed modifications. Methionine oxidation was set as a variable modification. The output was filtered using the Percolator algorithm with strict FDR set to 0.01. Quantification parameters included the allowance of unique and razor peptides, reporter abundance based on intensity, lot-specific isotopic purity correction factors, normalization based on total peptide amount, protein ratio based on protein abundance, and background-based hypothesis testing (t-test). Final protein list was analyzed for enrichment using STRING database version 11.5^66^ and ShinyGO 0.77^67^.

### Immunoblot analysis

Purified brain mitochondria were solubilized in Laemmli buffer and processed for Western blotting using standard procedures and Total OXPHOS Antibody Cocktail (#ab110411, Abcam). Briefly, solubilized protein was run on a 4–20% SDS-PAGE gel (Bio-Rad). After electrophoretic transfer to a PVDF membrane, Revert^™^ Total Protein Stain (Li-COR Biosciences) was applied to membrane per the manufacturers instructions. Membranes were blocked in Tris-Buffered Saline + 0.1% Tween 20 (TBS-T) containing 5% milk. For the primary antibodies, the mouse monoclonal MitoProfile Total OXPHOS Human WB antibody cocktail was diluted at 1:500 (abcam #ab110411). Other antibodies were diluted as recommended by manufacturers. Blots were visualized with Pierce^™^ ECL Western Blotting Substrate.

### Mouse perfusion and CNS Cryosectioning

Mice were perfused via the heart as described previously (PMID: 35129175). Briefly, animals were anesthetized with inhaled isoflurane prior to sequential cardiac perfusion with PBS (pH 7.4) followed by fresh 4% PFA dissolved in PBS (pH 7.4). The brain was removed and divided into right and left hemispheres via the longitudinal fissure. The spine was removed and divided into cervical and lumbar regions using the cervical and lumbar enlargements as landmarks. All tissues were cryoprotected in increasing concentrations of sucrose and embedded in Tissue-Tek OCT compound (Sakura Finetek USA #4583). Tissue sections were obtained using a Leica CM1950 cryostat. Lumbar cryosections were obtained at 30 mm, and stored in a cryoprotectant solution (20% glycerol, 30% ethylene glycol, 50% PBS pH 7.4) at −20°C. Individual brain hemispheres were sectioned coronally beginning at the olfactory bulb. 30 mm striatal sections were collected into cryoprotectant and stored at −20°C. 14 mm sections at the level of the Substantia Nigra Pars Compacta (SNc) were collected onto Superfrost plus slides (FisherScientific #12-550-15) in an alternating series of 4 slides, air dried and stored airtight at −70°C.

### Nissl staining and quantification

For Nissl staining, lumbar spines were thawed, washed in PBS and mounted onto Super frost plus slides and air dried. Slides were “defatted” overnight in a 1:1 mixture of absolute ethanol and chloroform. The next day, slides were hydrated through sequential immersions in absolute ethanol, 95% ethanol, and distilled water. A Coplin jar containing Cresyl Violet solution (Sigma-Aldrich #C5042) was warmed to 42°C and slides were incubated in this solution for 5 minutes. Slides were rinsed with distilled water before being dehydrated and cleared through sequential immersions in 95% ethanol, absolute ethanol, and xylene. Slides were mounted with Leica Micromount xylene based mounting medium (Leica Biosystems #3801731) and dried overnight. Whole slide scans were obtained at 20x using the Aperio AT2 slide scanner (Leica Biosystems). For image analysis, raw (“.svs”) files from the slide scanner were imported into QuPath (PMID 29203879). Motor neurons were counted in the anterior horn of the spinal cords in a blinded manner using criteria modified from Lee et. al. (PMID:16399671). Motor neurons were identified based on a large size of the soma (diameter greater than approximately 20mm), a pale staining round nucleus, and globular Nissl stain within the cytosol. Atrophic (chromatolytic) neurons were defined by a large size of the soma (diameter greater than approximately 20 mm), hypercondensation of nuclear material, and a pale-pink homogenous cytosol.

### Immunofluorescence of slide mounted sections

For anti- a-synuclein phospho S129 probing in the lumbar spine, sections were thawed, washed in PBS, mounted onto Super frost plus slides and air dried. Dried slides were washed in pH7.4 PBS + .05% Tween 20 (PBST) and incubated for one hour in blocking buffer (10% normal goat serum +.3% Triton-x-100 dissolved in PBST) at room temperature. Blocking buffer was discarded and slides were washed and incubated with primary antibody: rabbit anti-Alpha- synuclein (phospho S129) (Abcam #ab51253, 1:10000) diluted in blocking buffer overnight. Primary antibody was discarded, and slides were washed and incubated with secondary antibody: Alexa Fluor 633 conjugated goat anti-rabbit IgG (H+L) (Invitrogen #A-21070, 1:500) diluted in blocking buffer for 2 hours. Secondary antibody was discarded, and slides were washed and mounted with ProLong gold antifade mountant with DAPI (Invitrogen #P36391).

For Tyrosine Hydroxylase, and ubiquitin probing in the midbrain regions, slides corresponding to serial sections were thawed, washed in PBST, and incubated for one hour in blocking buffer at room temperature. Blocking buffer was discarded and slides were washed and incubated with primary antibody: chicken anti- Tyrosine Hydroxylase (Invitrogen #PA5–143583, 1:500), or rabbit anti- ubiquitin (Invitrogen #701339, 1:100) diluted in blocking buffer overnight. Primary antibody was discarded, and slides were washed and incubated with secondary antibody: Alexa Fluor 488 conjugated goat anti-chicken IgY (H+L) (Invitrogen #A11039, 1:500) or Alexa Fluor 633 conjugated goat anti-rabbit IgG (H+L) (Invitrogen #A-21070, 1:500) diluted in blocking buffer for 2 hours. Secondary antibody was discarded, and slides were washed and mounted with ProLong gold antifade mountant with DAPI (Invitrogen #P36391).

### Immunofluorescence of free floating sections

Free floating sections from the lumbar spine and striatum were thawed and washed in PBST prior to incubation for one hour in blocking buffer (10% normal goat serum +.3% Triton-x-100 dissolved in PBST) at room temperature. Blocking buffer was discarded and sections were washed and incubated with primary antibody overnight. Lumbar sections were incubated in rabbit anti- VAChT (Invitrogen #PA5–85782, 1:1000), mouse anti- GFAP (Cell Signaling Technology #3670, 1:2000), or rabbit anti- ubiquitin (Invitrogen #701339, 1:4000). Striatal sections were incubated with chicken anti- Tyrosine Hydroxylase (Invitrogen #PA5–143583, 1:400), or rabbit anti- ubiquitin (Invitrogen #701339, 1:1000). Primary antibody was discarded, and sections were washed and incubated with the appropriate secondary antibody: Alexa Fluor 633 conjugated goat anti- rabbit IgG (H+L) (Invitrogen #A-21070, 1:500), DyLight 550 conjugated goat anti- mouse IgG (H+L) (Invitrogen #84540, 1:500), or Alexa Fluor 488 conjugated goat anti- chicken IgY (H+L) (Invitrogen #A11039, 1:500) diluted in blocking buffer for 2 hours. Secondary antibody was discarded, and sections were washed before collection onto Super frost plus slides and mounting with ProLong gold antifade mountant with DAPI (Invitrogen #P36391).

### Detection of in situ apoptosis rate

The terminal deoxynucleotidyl transferase-mediated dUTP nick-end labeling (TUNEL) assay was used to quantify numbers of apoptotic cells within sections at the level of the midbrain. Slides were thawed and washed in pH7.4 PBS + .05% Tween 20 (PBST) prior to incubation for one hour in blocking buffer (10% normal goat serum +.3% Triton-x-100 dissolved in PBST). Blocking buffer was discarded and slides were washed prior to antigen retrieval via microwaving in a pH 6.0 Sodium Citrate Buffer (100mM Sodium Citrate + 3% Triton-x-100 dissolved in PBST). After reaching 86°C, slides were rapidly cooled in PBST, washed and incubated with the TUNEL reagent (Roche #12156792910) for 20 minutes in a light protected humidified chamber at 37°C. TUNEL reagent was discarded, and slides were washed before mounting with ProLong gold antifade mountant with DAPI (Invitrogen #P36391).

### Fluorescent image acquisition and signal quantification

To retain fluorescent signal strength, mounted slides were stored in the dark at 4°C and imaged within 72 hours for immunofluorescent probes, or within 24 hours for the TUNEL reaction. Z stack confocal images of the sections with or without tile stitching were obtained using the Leica SP8 confocal microscope. For image analysis, raw (“.lif”) Leica image files were imported into FIJI for processing. Where possible, automated image quantification was performed using custom built imageJ macros. The remaining images were quantified by a blinded observer using ImageJ.

To quantify the abundance and size of Alpha-synuclein phospho S129 puncta within the spinal cord, z stack images corresponding to the anterior horn of the spinal cord were obtained. Each z stack was analyzed via histogram to find the brightest focal plane. This focal plane was extracted and converted into an 8-bit image, to which an empirically determined threshold was applied. A pathology mask corresponding to Alpha-synuclein phospho S129 puncta was generated and quantified using the “analyze particles” function to generate individual puncta areas, puncta perimeters, and calculate the percentage of the field occupied by Alpha-synuclein phospho S129. To count the number of dopaminergic tyrosine hydroxylase (TH) positive cells within the Substantia Nigra Pars Compacta (SNc), tile-stitched z stacked images were analyzed manually by a blinded observer in ImageJ. A selection was drawn around the SNc to exclude the neighboring ventral tegmental area. Within this selection, TH positive cells from each focal plane were counted while partial cells were excluded. To count the number of motor neurons within the anterior horn of the spinal cord, max projected images of the VAChT signal were produced, from which motor neuron number was counted manually by a blinded observer. TH intensity within the striatum was calculated manually by a blinded observer using ImageJ quantification of the sum projection image.

To quantify the burden of ubiquitin inclusion bodies within regions of the midbrain at the level of the SNc, an imageJ macro was generated to calculate the size of individual puncta as well as the percentage of the field occupied by puncta. To accomplish this, the macro produced a max projection from the supplied tile-stitched z stacked image, and subsequently converted this projection into an 8-bit image. The midbrain reticular nucleus and the superior colliculus were identified as heavily affected regions within the end stage mice. A selection was drawn around each region and an empirical threshold was used to generate regional pathology masks. The “analyze particles” function was used to quantify the desired parameters from the pathology masks. A modification was made to the original macro to quantify the rate of TUNEL positive nuclei within the SNc. This modification allowed for the selection of the SNc, from which, parallel thresholding and particle analysis was performed to first count the number of nuclei within the field, and then count the number of TUNEL puncta above a size threshold within the field. To quantify GFAP pathology within the anterior horn of the spinal cord, z stacked images were analyzed via an imageJ macro which calculated the volume of GFAP pathology along the entire z stack via iterative empirical thresholding. From this macro, a volume of GFAP positive voxels was converted into metric volume using the image’s calibrated metadata.

### Central nervous system dissection

Neural tissues were dissected and snap-frozen as quickly as possible, and the spinal cord ejected from the spinal canal via hydraulic extrusion with ice cold DEPC-treated PBS. Accurate identification of the striatum was less straight forward than the cerebellum and spinal cord. First, cerebellar, brain stem, and olfactory bulb were removed from the forebrain. The brain was then cut sagittally along the interhemispheric fissure. Approaching from the medial aspect of the brain, inner brain material (including the thalamus, septum and underlying striatum) was removed by cutting just below the corpus collosum, thus separating these tissues from the hippocampus and associated cerebral cortex. The easily identifiable thalamus and hypothalamus were removed, and the remaining tissue from this “inner brain” material was labeled striatum. Tissue was frozen in liquid nitrogen.

### RNA purification and sequencing

Tissue was disputed in QIAzol lysis reagent, using the Qiagen TissueRuptor. RNA was then extracted using the Qiagen miRNeasy Mini Kit. RNA quality and quantity were assessed with the RNA 6000 Nano kit on the Agilent 2100 Bioanalyzer. For striatum, sequencing libraries were prepared using the Illumina TruSeq Stranded mRNA Library Prep kit, using 1ug total RNA as input. For cerebellum and spinal cord, sequencing libraries were prepared using the Illumina Stranded mRNA Library Prep kit Ligation, using 500ng Total RNA as input. The two library prep kits are equivalent. Library size was assessed with the DNA 1000 Kit on the Agilent 2100 Bioanalyzer. Libraries were quantified using the Quant-IT High Sensitivity dsDNA Assay (Invitrogen) on a Qubit 3.0 Fluorometer. Libraries were sequenced on the NextSeq 500 instrument, with paired end 2×75bp reads.

### RNAseq analysis

All analysis was performed on Partek Flow Genomic Analysis Software. All reads were aligned to the *mus musculus* genome (mm10) using STAR – 2.7.3a and quantified using Partek E/M. Reads were normalized with TPM (transcripts per million) and quantile normalization including all samples from spinal cord, cerebellum and striatum. We probed for differentially expressed genes using ANOVA, analyzing data from all tissues together. Within-tissue analyses shown as heat maps, volcano plots, and gene lists were generated in Partek from this analysis. Similarly, pathway analysis shown for the striatum data was performed using Partek Pathway Analysis software. For volcano plots, *Slc25a4* transcript was omitted to facilitate visualization.

### Behavioral Assays

Behavioral assays were performed in a particular order to minimize the likelihood that one test affects mouse behavior on subsequent days. The order went as follows: elevated plus maze, Y-maze spontaneous alternation test, open field activity test, novel object recognition test, Morris Water Maze, puzzle box test, followed by rotarod testing. Male and female mice were tested in all assays. Each data point shown in behavioral assays represents an independent mouse. Elevated plus maze, y-maze spontaneous alternation, and open field activity testing were done on two independent cohorts. For every test, mice were habituated to the testing room for 30 minutes prior to each test, and odors and residue was removed after each test with 70% ethanol. Mouse activity and scoring in each test was automatically measured using ANY-Maze behavioral tracking software, except for puzzle box, rotarod and beam walking tests, which were scored manually with the experimenter blinded to genotype. Elevated plus maze, y-maze, open field activity, and novel object recognition were performed as previously described^68^.

### Elevated plus maze (EPM)

EPM is used to assess anxiety-like behavior. We used a standard mouse EPM apparatus from San Diego Instruments, which consists of two closed arms and two open arms perpendicular to one another, forming a “plus” shape. Mice were placed in the center of the apparatus, facing an open arm, and allowed to explore freely for 5 minutes under ambient light. The time spent in the open arms is reported to reflect anxiety-like behavior, such that the less time in the open arms, the more anxiety-like the behavior is.

### Y-maze Spontaneous Alternation Test

We used the Y-maze Spontaneous Alternation Test to assess spatial working memory. A custom-built apparatus was used, which consisted of 3 walled arms 16 inches long that are angled 120° from one another. Mice were placed in the center and allowed to freely explore for 5 minutes in dim light. This test is based on the rodent’s tendency to explore new environments. Exploring the 3 arms consecutively, without re-entry into an arm, is called a triad. This implies that the mouse “remembers” which arm it was most recently in, despite all arms appearing identical. To control for difference in total number of arm entries, we report Fraction of Alternation, which is (total number of triads) / (total number of arm entries – 2). Mice with less than 5 total arm entries were excluded from Fraction of Alternation analysis.

### Open field activity test

Open field activity testing was performed to assess spontaneous locomotor activity and anxiety-like behavior. We used a standard apparatus from San Diego Instruments, which consists of 50 cm × 50 cm open field surrounded by non-transparent walls. Mice were allowed to explore freely for 10 minutes in ambient light. The total time spent away from the pre-designated “center zone” is reported to reflect anxiety-like behavior.

### Novel object recognition (NOR) test

NOR testing was performed to assess long-term object recognition memory and was performed in the open field apparatus. Briefly, two identical objects (cubes) were placed in the chamber and the mice were allowed to explore the objects for 5 minutes per day in dim lighting for two training days. On the third day, a novel object (cylindrical piece of wood) replaced one of the cubes, and mice were allowed to explore the chamber for 10 minutes. If the mice remember the objects from the training days, then they tend to spend more time exploring the novel object. Reported is the time spent interacting with the novel object on the final day of testing. Mice with less than 5 seconds of total object exploration time on the final testing day were excluded from discrimination index analysis.

### Morris water maze

Morris water maze was performed to assess long-term spatial memory and learning, and was performed in an inflatable hot tub with the water temperature at 26°C in dim lighting, essentially as previously described^69^. Briefly, mice were placed in the circular pool for 4 trials per day and allowed one minute to find a platform that they can stand on. Platform was made of transparent plastic and placed just beneath the water surface as to make it invisible to the rodents. For pre-training day 1, mice were placed on the platform for one minute, followed by placement of the mouse proximally to the platform so it learns it can escape the water. On pre-training day 2, mice were placed in a pseudo-randomized quadrant of the pool and allowed one minute to escape to the platform, which was made visible on this day with a small flag. Then, for 7 training days, the platform was below the surface with no flag, and the mice were given 4 trials per day to learn where the platform was using spatial cues from around the room. On the probe day, the day after training day 7, the platform was removed, and the amount of time the mice spent swimming in the quadrant of the pool that previously had the platform, as well as the number of times the mice entered the platform zone, were recorded. The better long-term spatial memory the rodent has, the more time it spends in the target quadrant, and the more times it will enter the platform zone.

### Puzzle Box Test

To assess executive function, we performed the puzzle box test as previously described^23,24^. Briefly, mice were placed in a custom-built 60 cm × 28 cm apparatus that is well-lit with a small fan blowing into it to create a stressful environment with multisensory stimuli. The mice are given a passage (5 cm by 5 cm doorway) to small and dark target location filled with home bedding to make the mice comfortable. The idea is to test the mouse’s ability to “problem solve” by removing obstacles between the stressful environment and the target location. In condition 0, there is no obstacle. Condition 1 had a U-shaped channel within the doorway. Condition 2 had the same U-shaped channel within the doorway, but with a mound of cage bedding placed in it to block the doorway. Condition 3 had a crumped piece of paper placed in the doorway. Finally, condition 4 had a cube placed in the doorway, with a raised edge on top of it such that the mice had to pull it out of the doorway and could not push it through. We manually recorded the time it took to reach the target location and plotted that as “escape latency”. A maximum of 5 minutes was allowed for each trial.

### Beam walking test

Beam walking test was used as a measure of balance and coordination. Although typically the primary read-out for this assay is time to traverse the beam, analysis of these data was compromised by the substantially increased locomotor activity of transgenic a-synuclein^A53T^ mice. Thus, we prioritized the number of times each mouse’s hind limbs slipped off the beam as the primary read-out for balance and coordination.

We custom-built a beam walking apparatus, consisting of a one-meter-long wooden beam suspended ~16 inches above the floor. The start end of the beam was suspended in the air without an escape route, and the other end has the target box for the mice to escape into. The target box is an enclosed black cube of about 9 inches on each side. Before each test, home bedding from the tested mouse is added to the escape box for enticement. Aversive stimuli (a bright LED light and a small fan) were placed at the start end of the beam such that the escape box was the only route to protection. Mice were trained on an easy beam (0.5-inch rectangle) over two days. In training, if a mouse stopped moving while on the beam it was gently prodded by the experimenter. On training day 1, the mouse was placed directly in the target box for one minute, then placed on the beam two inches from the target box and allowed to escape to the target box, and finally placed at the far end of the beam for full traverse of the beam. On training day 2, each mouse was given 3 more trials on the easy beam. On test day, each mouse was tested twice on 0.5-inch circular and 0.25-inch rectangular beams. The number of hindlimb slips per beam was manually scored by an experimenter who was blinded to genotype. Plotted in Figure 4 is the average number of slips of the two trials.

### Accelerating rotarod test

Mice were initially acclimated to the rotarod for 30 seconds at 4 rotations per minute (rpm). Mice aged 19–22 months (n > 16 mice per genotype, n > 5 per sex per genotype) were tested on a high difficulty rotarod protocol of acceleration from 4 to 40 rpm over 2 minutes, three trials per day for two consecutive days. The time at which the mouse fell off the rod was recorded and plotted with the experimenter blinded to genotype.

### Treadmill exhaustion test

Treadmill tests were performed on an Exer 3/6 animal treadmill at a 5° incline (Columbus Instruments). For one week, mice were familiarized with the treadmill with 10-minute running sessions at 10 m/min every other day. For resistance testing, the treadmill started at 9 m/min with 1.2 m/min/min acceleration until the mice reached exhaustion. For power testing, the treadmill started at 9 m/min with 1.8 m/min/min acceleration until the mice reached exhaustion. Mice were deemed exhausted when they remained in continuous contact with the shock grid for 5 seconds. Three full days of rest were allowed between training and each testing day.

### Optomotor response test

Optomotor acuity and contrast sensitivity of mice were determined by observing their optomotor behavior responses to a rotating sine wave grating stimulus using the OptoMotry© system^70^ as described previously^71,72^. Briefly, mice were placed on a pedestal at a center of the OptoMotry chamber enclosed by four computer monitors. The computer randomly displayed sinusoidal pattern gratings rotating in a clockwise or counter-clockwise direction. The observer was blind to the direction of rotation and chose the direction of pattern rotation based on the animal’s behavior. Auditory feedback indicated to the observer whether the selected direction was correct or incorrect. Using a staircase paradigm, the computer program controlled the spatial frequency and contrast of the stimulus. The threshold was set at 70% correct responses. Contrast sensitivity was defined as the reciprocal of the threshold contrast value and was measured at 1.5 Hz and a spatial frequency of 0.128 cycles/degree. Acuity was measured at a speed of rotation of 12 degrees/s and 100% contrast. All measurements were performed at the unattenuated maximal luminance of the OptoMotry© system (~70 cd/m^2^, producing approximately 1500 R*/rod/s^73^).

### Statistical Analysis

Statistical analyses were performed using GraphPad Prism. For behavioral tests, we always probed for a statistically significant effect of Genotype, Sex and Genotype × Sex interactions. If data are presented without separating sex, this indicates there was no significant main effect of sex and no significant Genotype × Sex interaction. For details on statistical testing of specific data, please see Figure Legends.

## Supplementary Material

Supplementary Files

This is a list of supplementary files associated with this preprint. Click to download.

- SupplementaryTable1SC.xlsx
- SupplementaryTable2Cerebellum.xlsx
- SupplementaryTable3Striatum.xlsx
- SupplementaryTable4SCinsolproteomics.xlsx
- SupplementaryTable5SCsoluableproteomics.xlsx
- NCOMMS2328432Ars.pdf
- CoyneetalSupplementaryInformation.docx
- Alluncroppedimages.pdf

## Data Availability

The RNA-Seq and proteomic datasets generated as part of this study are presented as supplemental tables. The data have also been deposited to NCBI Gene Expression Omnibus/Sequence Read Archive with the accession numbers GSE236975 (reviewer access token: uzyzukgandydpid) The mass spectrometry proteomics data have been deposited to the ProteomeXchange Consortium via the PRIDE [1] partner repository with the dataset identifier PXD043657 and 10.6019/PXD043657. Reviewer Account details: (URL: https://www.proteomexchange.org/; Username: reviewer_pxd043657@ebi.ac.uk; Password: L7i5qgC1) There was no code generated as part of this study.
